# New Statin-Safety Reassurance: How Far Can It Extend to South Asian Patients?

**DOI:** 10.7759/cureus.116538

**Published:** 2026-09-19

**Authors:** Jay Maradia

**Affiliations:** 1 Pharmacology, HBT Medical College And Dr. RN Cooper Municipal General Hospital, Mumbai, IND

**Keywords:** adverse drug events, management of cholesterol, pharmaco-vigilance, south asian, statin safety

## Abstract

Statin product labels list many potential adverse effects, and concern about these effects is a common reason why patients stop therapy or never start it, despite the well-established cardiovascular benefit of statins. This editorial argues that any reassurance about statin safety should be extended to South Asian patients only with caution, since their statin pharmacokinetics may differ from those of the populations from which most safety evidence is drawn. We treat this as a reasonable hypothesis worth testing through subgroup-level data, not as a settled limitation. We call for statin labelling to be revised based on solid evidence, including ethnicity-stratified data where it shows a real difference in safety or efficacy, rather than through a blanket relaxation of adverse-effect warnings.

## Editorial

Statins are among the most widely prescribed drugs worldwide, used for both primary and secondary prevention of atherosclerotic cardiovascular disease. Yet concern among patients about adverse effects remains a major reason why people never start statins or stop them prematurely. The Cholesterol Treatment Trialists' (CTT) Collaboration recently published an individual-participant-data meta-analysis in The Lancet that offers the most rigorous evidence to date on this question. It deserves close attention from prescribers in high-risk, resource-limited settings such as India [1].

The CTT Collaboration pooled data from 19 double-blind, placebo-controlled statin trials, which enrolled 123,940 participants and had a median follow-up of 4.5 years. It also included four trials comparing more-intensive with less-intensive statin regimens, which enrolled another 30,724 participants. Together, these allowed the collaboration to test 66 undesirable effects taken directly from statin product labels, none of which it had assessed before. After correcting for multiple testing at a 5% false discovery rate, only four of these 66 undesirable effects showed a statistically significant excess with statin therapy: abnormal liver transaminases (0.30% per year with statin vs 0.22% with placebo, an absolute excess of 0.09% per year), other abnormal liver function tests (0.25% vs 0.20%, absolute excess 0.05% per year), altered urinary composition (0.21% vs 0.18%, absolute excess 0.03% per year), and oedema (1.38% vs 1.31%, absolute excess 0.07% per year). Within these randomised trials, no statistically significant excess was seen for cognitive impairment, depression, sleep disturbance, peripheral neuropathy, erectile and sexual dysfunction, or most other effects listed on statin package inserts worldwide. This work builds on, rather than repeats, the collaboration's earlier findings of genuine, modest excess risks for muscle symptoms [2] and new-onset diabetes [3]. Both had already been established, so they were excluded from this 66-outcome analysis [1].

This is a methodologically strong piece of work. Individual patient-level data, double-blind comparators, and strict correction for multiple testing all reduce the confounding and detection bias that can affect the observational and spontaneous-reporting data feeding into product labelling, which also draws on other sources such as pre-approval trials and pharmacokinetic studies. An accompanying commentary makes a fair point: this kind of recalibration is overdue for some labelled effects. Once a term has entered a product label, it is not always re-checked when good randomised evidence becomes available [4].

Despite the correspondence this analysis has already generated [5], two questions still matter for prescribing practice in India and other South Asian settings. First, the trials contributing to the CTT Collaboration analysis have historically enrolled most of their participants in Europe, North America, and Australia. The paper does report race and ethnicity for participants in the trials that collected this information, yet its adverse-effect findings do not appear to be broken down by ethnic subgroup. It is well recognised that South Asian populations tend to develop coronary artery disease earlier and more often than European populations, and regulatory dose ceilings for simvastatin and rosuvastatin in Asian populations already reflect known differences in drug exposure. Taken together, this raises a reasonable hypothesis, not yet directly tested, that the safety profile in this meta-analysis may not translate uniformly to South Asian patients specifically. That hypothesis is worth testing formally, through future CTT data-sharing work or a dedicated South Asian pharmacovigilance cohort, rather than being treated as an established gap in the current findings.

Second, India's Pharmacovigilance Programme and the Central Drugs Standard Control Organisation largely inherit product label content from the original manufacturer's labelling and rarely reassess it independently against new randomised evidence. If official labels really do overstate non-hepatic, non-muscular adverse effects, this cuts both ways. Toning down nocebo-driven symptom reporting could meaningfully reduce unwarranted statin discontinuation in a population where cardiovascular risk is already high, and statin use is already low. But if clinicians adopt a blanket reassurance message without paying attention to the smaller set of effects that this analysis and its predecessors have confirmed, mainly myopathy and new-onset diabetes [2,3], they risk under-counselling patients who are genuinely at risk.

We would welcome a labelling revision process for India and similar settings that is evidence-based rather than a wholesale relaxation of the adverse-effect list. Any move toward ethnicity-stratified labelling should rest on solid evidence of a real difference in safety or efficacy, not on caution alone, and it should stay alert to the handful of effects this body of work has genuinely confirmed. Until that evidence exists, clinicians should use this reassurance carefully. This reassurance can help counter unwarranted nocebo-driven non-adherence. Baseline liver function testing, with further testing only as clinically indicated, already reflects current regulatory guidance and is not a recommendation specific to this analysis. Continued attention to muscle symptoms remains warranted, consistent with the CTT Collaboration's own findings [1,2].
